# Characterization of the complete mitochondrial genome of Golden cusk, *Sirembo imberbis* (Ophidiiformes:Ophidiidae)

**DOI:** 10.1080/23802359.2020.1840942

**Published:** 2020-12-24

**Authors:** Nazia Tabassum, Yeonghye Kim, Hyun-Woo Kim

**Affiliations:** a Department of 4th Industrial Convergence Bionix Engineering, Pukyong National University, Busan, Republic of Korea; b Fisheries Resources Management Division, National Institute of Fisheries, Gijang-gun, Republic of Korea; c Department of Marine Biology, Pukyong National University, Busan, Republic of Korea

**Keywords:** Cusk eel, NGS, Ophidiidae, *Sirembo imberbis*, mitochondrial genome

## Abstract

The complete mitochondrial genome of *Sirembo imberbis* was determined by the bioinformatic assembly of the next generation sequencing (NGS) reads. Total length of the mitogenome was 16,717 bp, which harbors the conserved 13 protein-coding genes (PCGs), 2 ribosomal RNAs (12S and 16S), 22 tRNAs, and two non-coding region (the control region and the origin of light-strand replication). Among 13 protein-coding genes, unusual start codon (GTG) was exclusively identified in COX1, while the incomplete stop codons (TA- or T–) were detected in COX2, COX3, ND2, ND3, ND4 and CytB. As a result of phylogenetic tree, *S. imberbis* formed a cluster of the family Ophidiidae together with *Bassozetus zenkevitchi* and *Lamprogrammus niger*.

## Introduction

*Sirembo imberbis* (Siebold et al. 1844) is one of the five recognized species in this genus *Sirembo*, commonly known as the cusk-ells. This species can be distinguished from the other four Sirembo species by additional horizontal rows of dusky blotches on the body (Nielsen et al. 2015). They are widely distributed in the Pacific from Korea to the northwestern Australia and the genetic information is required for its scientific management and conservation.

*Sirembo imberbis* was collected from the southern coastal water of Korean peninsula (34°21′25.7″N 128°22′37.9″E) during the research survey funded by the Ministry of Oceans and Fisheries. The identity of the specimen was confirmed by COI sequence identity (JQ681432) as well as its morphological characteristics. The specimen and its DNA are stored at the Marine Biodiversity Institute of Korea (MABIK GR00004105). Mitochondrial DNA was extracted by a commercial DNA isolation kit (Abcam, UK), which war further sheared by Covaris M220 Focused-Ultrasonicator (Covaris Inc., San Diego, CA, USA). TruSeq® RNA library preparation kit V2 was used to prepare a library for MiSeq sequencing platform (Illumina, San Diego, CA, USA). Assembly of raw reads and gene annotation were performed by Geneious^®^ 11.0.2 software (Kearse et al. 2012). The loci and structures of 22 tRNAs were predicted by tRNAScan-SE software (Lowe and Chan 2016). A phylogenetic tree was built with a minimum evolution (ME) algorithm using MEGA7.0 programs (Kumar et al. 2016).

The complete mitogenome of *S. imberbis*(MN937450) was 16,717 bp in length, which contained canonical eukaryotic 37 genes. Proportions of (A + T) was 57.1%, which was slightly higher than those of G + C (42.9%). Among 13 PCGs, only ND6 was encoded on light (L) strand, while the other 12 were on light (L) strand. Unusual start codon (GTG) was exclusively identified in COX1, while the incomplete stop codons (TA- or T–) were detected in COX2, COX3, ND2, ND3, ND4 and CytB. All 22 transfer RNAs (tRNAs) were folded into a typical clover-leaf molecular structureexcept the tRNA^Ser-GCT^, which lack D-arm. Two ribosomal RNAs (12S and 16S rRNAs) were encoded on H-strand. The origin of light-strand replication (OL) was predicted within a cluster of WANCY tRNAs, while the hypervariable control region (D-loop) was found between CytB and 12S rRNA.

**Figure 1.**
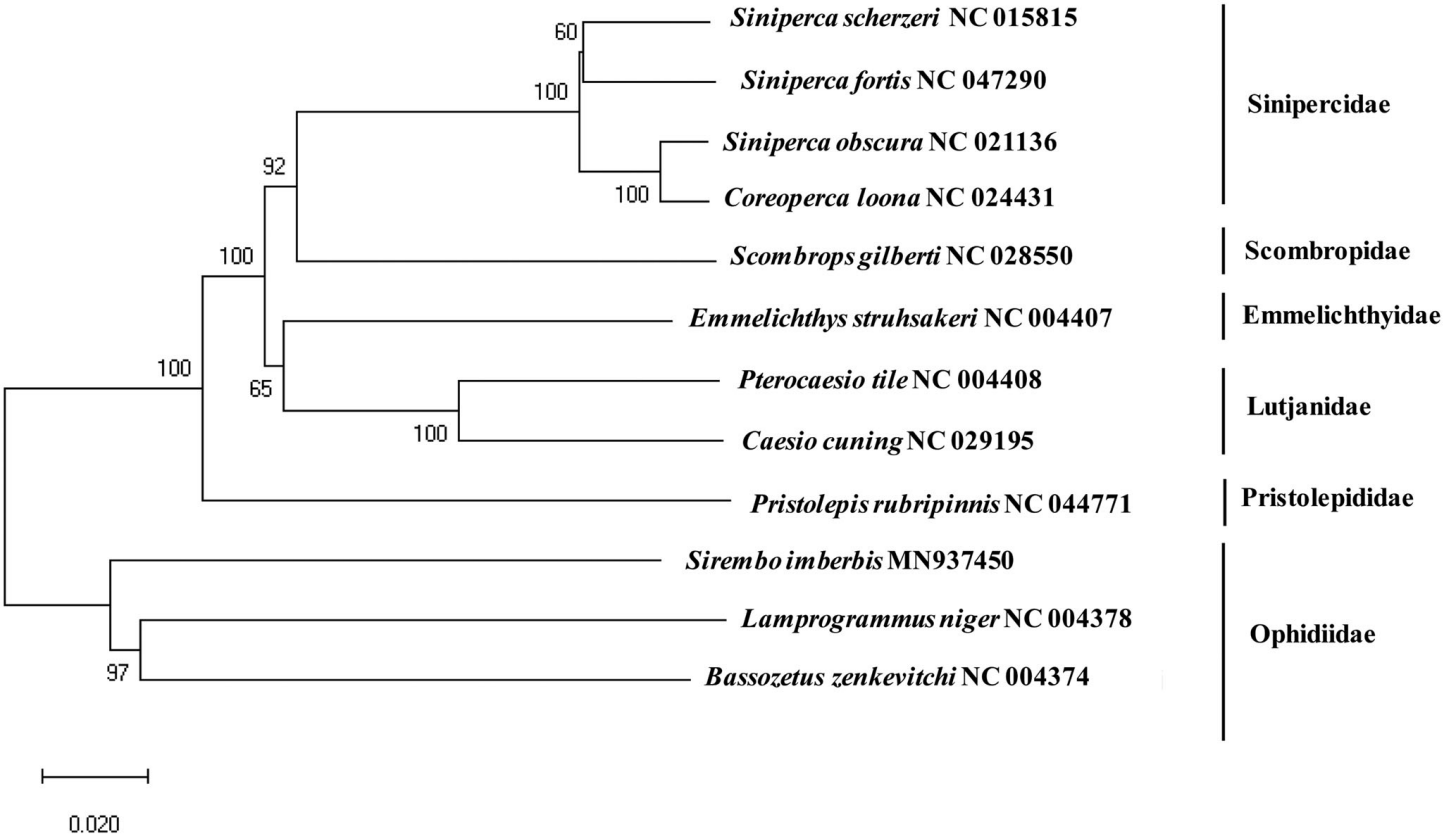
Phylogenetic construct of *Sirembo imberbis*among the fish order Perciformes: a phylogenetic tree was constructed with the currently reported complete mitochondrial genome in the order Perciformes by using MEGA7 software using Minimum Evolution (ME) algorithm with 1,000 bootstrap replications. GenBank accession numbers is followed by scientific name by each species.

A phylogenetic tree was constructed to identify the evolutionary relationship of *S. imberbis* with other currently reported fishes of order Perciformes (Figure 1). Since it is the first report of the complete mitogenome sequence in the genus Sirembo, *S. imberbis* (MN937450) formed a cluster of the family Ophidiidae together with its relative species, including *Bassozetus zenkevitchi* (81.62%) and *Lamprogrammus niger* (81.13%). According to the fishbase (http://www.fishbase.org/), there are approximately 240 species in the family and additional mitogenome sequences should be supplemented for the better understanding of their evolutional relationship.

## Data Availability

The data that support the findings of this study are available in GenBank database at *Sirembo imberbis*(GenBank Number: MN937450) https://www.ncbi.nlm.nih.gov/nuccore/MN937450.1
